# Partial response to pralsetinib in an advanced pulmonary sarcomatoid carcinoma patient harboring a *KIF5B-RET* rearrangement: a case report

**DOI:** 10.1186/s12957-022-02848-z

**Published:** 2022-12-06

**Authors:** Ying Wu, Zhecheng Yan, Juan Pan, Xiaona Chang, Bo Huang, Danju Luo, Rui Meng, Heshui Shi, Jun Fan, Xiu Nie

**Affiliations:** 1 Department of Pathology, Union Hospital, Tongji Medical College, Huazhong University of Science and Technology, Wuhan, 430022 Hubei China; 2 Cancer Center, Union Hospital, Tongji Medical College, Huazhong University of Science and Technology, Wuhan, China; 3 Department of Radiology, Union Hospital, Tongji Medical College, Huazhong University of Science and Technology, Wuhan, 430022 Hubei China

**Keywords:** Pulmonary sarcomatoid carcinoma, *KIF5B-RET* gene fusion, Pralsetinib

## Abstract

**Background:**

Pulmonary sarcomatoid carcinoma (PSC) is a rare and unconventional non-small-cell lung cancer (NSCLC) that appears to be aggressive, with a poor prognosis and response to conventional treatment. Approximately 30% of PSCs have potentially targetable genomic alterations, but few studies have involved *RET* gene fusions, and corresponding targeted therapies are lacking.

**Case presentation:**

In this report, we describe a patient with PSC harboring a *KIF5B-RET* gene fusion who was initially diagnosed with stage IVb lung cancer. Due to the poor performance status, the patient was unable to tolerate any radiotherapy or chemotherapy. Based on the next-generation sequencing (NGS) result of *RET* gene fusion, the patient was treated with pralsetinib. Two months after the treatment, the patient achieved a partial response.

**Conclusions:**

Our case indicates that *RET* is one of the main driver oncogenes of PSC and provides useful information for precise *RET* inhibitor administration in the future. Thus, the use of comprehensive genomic profiling may provide important treatment options for PSC.

## Introduction

Pulmonary sarcomatoid carcinoma (PSC) is a rare and highly invasive tumor with an extremely low incidence of less than 1% in all lung cancers [1–3]. In accordance with the 2021 World Health Organization (WHO) classification of lung tumors, PSC, a poorly differentiated non-small-cell lung cancer (NSCLC), can be divided into five subtypes: pleomorphic carcinoma, spindle cell carcinoma, giant cell carcinoma, carcinosarcoma, and pulmonary blastoma [4]. Compared with other NSCLC subtypes, patients with PSC have a more aggressive clinical course and a poorer prognosis with the 5-year overall survival (OS) ranging between 10 and 21% [5–7].

Currently available treatment options for PSC are limited due to resistance to chemotherapy, low responsiveness to radiotherapy and extremely quick recurrence after surgical resection [8–11]. Schorock et al. demonstrated that approximately 30% of PSCs are accompanied by potentially targetable genomic alterations, providing a comprehensive genomic understanding for developing targeted therapeutic strategies [12]. The most frequently mutated genes across different studies include *TP53*, *KRAS*, *CDKN2A*, *PTEN*, *MET*, *EGFR*, *BRAF*, and *HER2* [12–14]. In a cohort of PSC cases, 79% (44/56) of the patients harbored mutations in *TP53*, and 57% of the patients harbored mutations in genes of the receptor tyrosine kinase (RTK)/RAS pathway: *EGFR* (16%), *KRAS* (14%), *MET* (13%), *BRAF* (7%), *NF1* (5%), and *NRAS* (4%) [14]. Schorock et al. revealed a 0.8% (1/12) *RET* proto-oncogene amplification in PSC [12], and Liang et al. found two PSC patients (2/32) with RET fusions, *KIF5B-RET* and *TUBD1-RET* [13]. Nevertheless, detailed treatments of PSC patients with RET alterations were not provided in either of these studies. Here, we report a case of PSC with a *KIF5B-RET* fusion that exhibited a remarkable response to the selective RET inhibitor pralsetinib.

## Case presentation

**Fig. 1:**
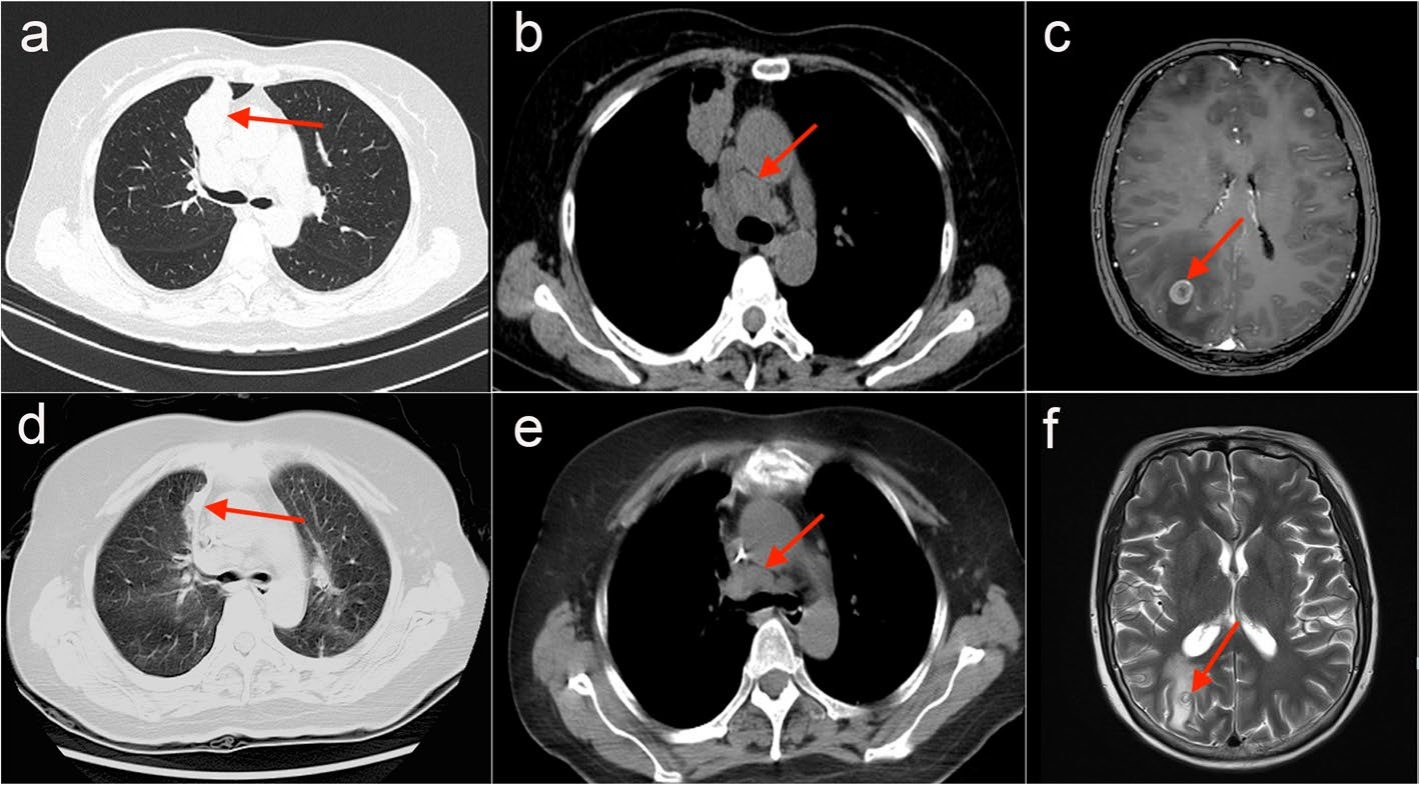
The image changes before and after treatment. The CT scan before treatment showed the mass in the right upper lobe sized 4.6 × 3.2 cm (**a**), mediastinal 4R enlarged lymph nodes (**b**). Contrast enhanced MRI of brain before treatment revealed multiple intracranial space-occupying lesions (**c**). The CT examination after treatment with pralsetinib displayed the mass sized 2.8 × 1.2 cm in the right upper lobe (**d**), marked reduction in the size of lymph node in mediastinum 4R (**e**). Brain MRI after treatment demonstrated the shrink of the right parieto-occipital region (**f**). Red arrows indicate the tumor or lymph node lesions

**Fig. 2:**
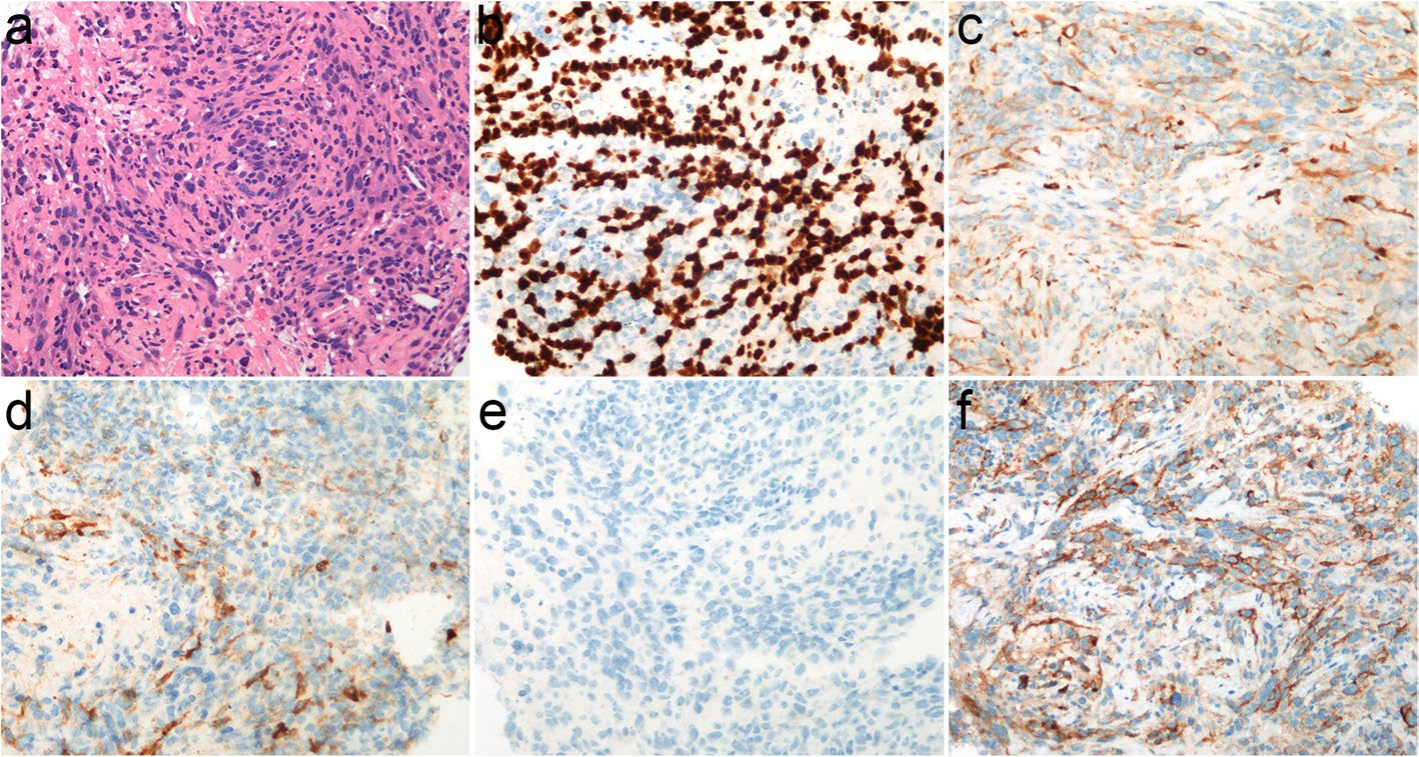
Microscopic images of the PSC. **a** The tumor demonstrated a spindle cell-like morphology with poor differentiation (× 200). **b** The tumor cells showed a positive nuclear signal for TTF-1 (× 200). **c** The tumor cells showed a weakly positive cytoplasmic signal for PCK (× 200). **d** The tumor cells showed a weakly positive cytoplasmic signal for EMA (× 200). **e** The tumor cells showed a negative cytoplasmic signal for P40 (× 200). **f** PD-L1 TPS immunohistochemistry analysis showed at least 60% (× 200)

**Fig. 3:**
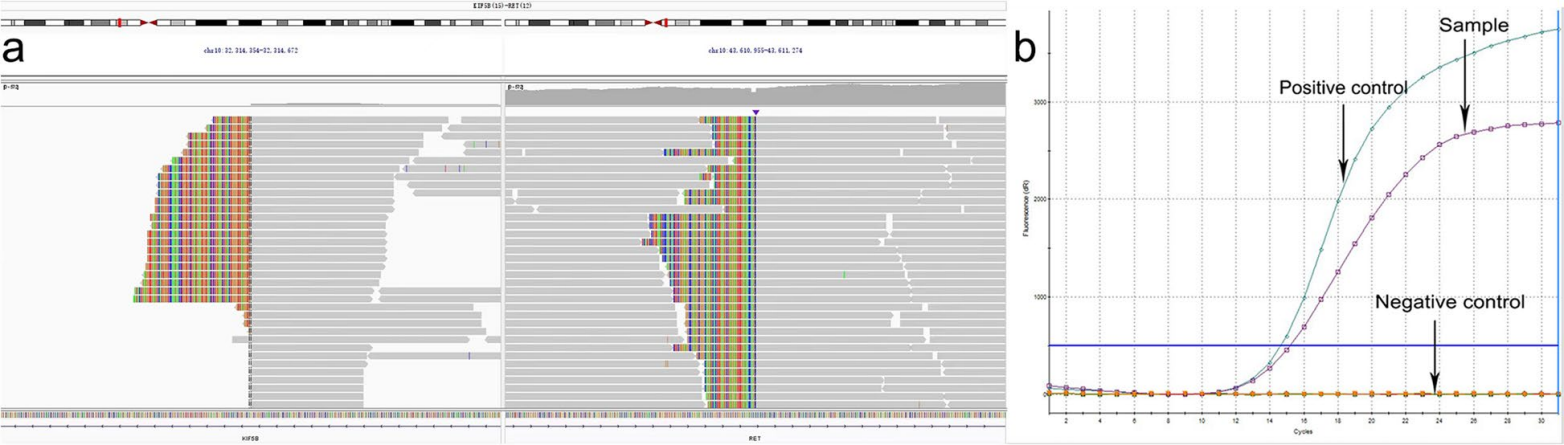
Detection of the KIF5B-RET gene fusion in the current case. **a** NGS revealed the presence of the KIF5B-RET gene fusion (3.05% abundance in tissue). **b** KIF5B-RET gene fusion was detected by ARMS RT–PCR

A 52-year-old nonsmoking female was admitted to our hospital due to cough and bilateral low back pain for one month. A chest computed tomography (CT) scan showed a mass in the right upper lobe (4.6 × 3.2 cm) (Fig. 1a) and multiple enlarged lymph nodes in the mediastinum 4R (Fig. 1b), mediastinum and bilateral axillary; a small nodule (1.0 × 0.6 cm) was seen in the left upper lobe; and there was a pathological fracture of the 12th thoracic (T12) vertebral body. Cranial magnetic resonance imaging (MRI) showed multiple intracranial space-occupying lesions (Fig. 1c), considering lung cancer with multiple metastases to the brain, bone and lymph nodes (clinical disease stage: IVb, cT2N2M1c). After the statement of informed consent, CT-guided percutaneous needle biopsy of the lung mass was performed. Microscopically, the tumor cells were mostly poorly differentiated with an almost spindle cell-like morphology (Fig. 2a). On immunohistochemistry, tumor cells were positive for TTF-1 (Fig. 2b) and vimentin, weakly positive for PCK (Fig. 2c) and EMA (Fig. 2d), and negative for P40 (Fig. 2e), SMA, S100 and desmin. Eventually, sarcomatoid carcinoma (spindle cell carcinoma) was diagnosed. The tumor proportion score (TPS) of programmed cell death ligand 1 (PD-L1) expression was 60% (Fig. 2f). DNA-based next-generation sequencing (NGS) revealed the presence of the *KIF5B (15)-RET (12)* fusion (3.05% abundance in tissue) (Fig. 3a), which was verified by ARMS RT–PCR assay (Amoy Diagnostics, Xiamen, China) (Fig. 3b). Due to the poor performance status and severe intestinal obstructive symptoms, the patient was unable to tolerate any radiotherapy or chemotherapy. On the basis of her *RET* fusion status, RET tyrosine kinase inhibitor treatment with pralsetinib, 125 mg three times daily, commenced on January 24th, 2022. Two months after initiation of the treatment, the examinations showed an excellent partial response, including the significant reduction of the mass (2.8 × 1.2 cm) in the right upper lobe (Fig. 1d), the marked decrease in the size of lymph nodes in the mediastinum 4R (Fig. 1e) and the shrink of the metastatic lesion in the right parieto-occipital region (Fig. 1f). And the clinical symptoms were relieved. The patient remains under follow-up.

## Discussion

PSC is a unique subtype of NSCLC with an exceptionally poor prognosis and resistance to traditional chemotherapy. However, oncogenic mutations, fusions, and copy number alterations of driver oncogenes specified in the NCCN NSCLC guidelines have been identified in 30% of cases. In recent years, a higher frequency of *MET* exon 14 splicing site mutations has been reported in PSC, with a prevalence ranging from 4.9% to 31.8%, compared to 2.62% in all NSCLC. With limited reports, the frequency of *MET* amplification in PSC ranges from 4.8 to 13.6%, while the *MET* protein overexpression rate in PSC ranges from 17 to 40.9% [15–19]. Lu et al. demonstrated that the objective response rate (ORR) of savolitinib in PSCs harboring *MET* exon 14 splicing site mutations was 40.0% (10/25) [20]. In addition, Ann Valter et al. reported a case of PSC harboring the *ALK-EML4* fusion gene that displayed a good response to crizotinib [21]. Moreover, in the report by Zou et al., a PSC patient with an *EGFR* exon 21 L858R gene mutation was successfully treated with erlotinib after failing chemoradiotherapy and remained progression-free for 6 months [22].

To date, *RET* rearrangement has been identified in approximately 1–2% of NSCLC patients, involving the most common *RET* fusions: *KIF5B-RET* (70–90%) and *CCDC6-RET* (10–25%), followed by *NCOA4-RET*, *TRIM33-RET*, *ZNF477P-RET*, *ERCC1-RET*, *HTR4-RET*, and *CLIP1-RET* (18%) [23–26]. Specifically in PSC, *RET* amplification was reported by Schorock et al. [12], and *KIF5B-RET* along with *TUBD1-RET* fusion was identified by Liang et al. [13]. Even so, neither study provided treatment details for PSC patients with *RET* alterations. Currently, drugs such as selpercatinib and pralsetinib are FDA-approved RET kinase inhibitors for the treatment of NSCLC. The clinically important effects on the overall response rate (ORR) of selpercatinib were observed in a multicenter, open-label, multicohort clinical trial (LIBRETTO-001, NCT03157128) in patients whose tumors had *RET* alterations. ORRs within *RET* fusion–positive NSCLC patients were 64% in prior platinum-treated patients and 85% in treatment-naive patients [27]. In addition, pralsetinib has recently been reported to be a new, well-tolerated, promising treatment option for RET fusion-positive NSCLC patients, with an ORR ranging from 61% (prior platinum-treated patients) to 70% (treatment-naive patients) (ARROW, NCT03037385) [28]. Nevertheless, neither of these studies explicitly stated that PSC was involved.

To our knowledge, this is the first case report describing a clinical response to pralsetinib in a patient with PSC harboring a *KIF5B-RET* fusion, which demonstrates that *RET* is one of the main driver oncogenes of PSC and is sensitive to matched targeted therapy. Furthermore, comprehensive genomic profiling may provide important treatment options for a historically poorly characterized and difficult-to-treat disease.

## Data Availability

Data sharing does not apply to this article as no datasets were generated to analyzed during the current study.

## Abbreviations

PSC: Pulmonary sarcomatoid carcinoma
NSCLC: Non-small-cell lung cancer
NGS: Next-generation sequencing
WHO: World Health Organization
OS: Overall survival
CT: Computed tomography
ORR: Objective response rate
